# Maintenance Energy Requirements of Double-Muscled Belgian Blue Beef Cows

**DOI:** 10.3390/ani5010089

**Published:** 2015-02-13

**Authors:** Leo O. Fiems, Johan L. De Boever, José M. Vanacker, Sam De Campeneere

**Affiliations:** ILVO—Animal Sciences Unit, Scheldeweg 68, B-9090 Melle, Belgium; E-Mails: johan.deboever@ilvo.vlaanderen.be (J.L.D.B.); jose.vanacker@ilvo.vlaanderen.be (J.M.V.); sam.decampeneere@ilvo.vlaanderen.be (S.D.C.)

**Keywords:** double-muscled, beef cows, energy, maintenance requirement

## Abstract

**Simple Summary:**

Double-muscled Belgian Blue animals are extremely lean, characterized by a deviant muscle fiber type with more fast-glycolytic fibers, compared to non-double-muscled animals. This fiber type may result in lower maintenance energy requirements. On the other hand, lean meat animals mostly have a higher rate of protein turnover, which requires more energy for maintenance. Therefore, maintenance requirements of Belgian Blue cows were investigated based on a zero body weight gain. This technique showed that maintenance energy requirements of double-muscled Belgian Blue beef cows were close to the mean requirements of cows of other beef genotypes.

**Abstract:**

Sixty non-pregnant, non-lactating double-muscled Belgian Blue (DMBB) cows were used to estimate the energy required to maintain body weight (BW). They were fed one of three energy levels for 112 or 140 days, corresponding to approximately 100%, 80% or 70% of their total energy requirements. The relationship between daily energy intake and BW and daily BW change was developed using regression analysis. Maintenance energy requirements were estimated from the regression equation by setting BW gain to zero. Metabolizable and net energy for maintenance amounted to 0.569 ± 0.001 and 0.332 ± 0.001 MJ per kg BW^0.75^/d, respectively. Maintenance energy requirements were not dependent on energy level (*p* > 0.10). Parity affected maintenance energy requirements (*p* < 0.001), although the small numerical differences between parities may hardly be nutritionally relevant. Maintenance energy requirements of DMBB beef cows were close to the mean energy requirements of other beef genotypes reported in the literature.

## 1. Introduction

Improving the efficiency of energy utilization is of paramount importance from several points of view. First, a higher efficiency means a reduction of feed costs for livestock production. This may result in an increased income for the farmer. Furthermore, a higher efficiency also means that nutrients are better utilized by the animal, resulting in a lower excretion into the environment and lower costs for manure management. Feeding an increasing world population up to 9 billion people [1], or more, also means that the competition between feed and food will increase in the future. Nowadays, a substantial part of livestock is fed on grain and other plants that could be used as human food. So, an efficient animal nutrition is a key factor to reduce the environmental load from animal production and the competition with human food. Furthermore, land use for feed production may also interact with land use for bio-fuel production.

Double-muscled Belgian Blue (DMBB) animals originated from the dual-purpose Belgian Blue cattle breed. Due to an intensive selection, there was a transition from halfway the fifties to the end of the sixties of the previous century to animals with a larger muscular development [2]. In 1973 the Belgian Blue breed was divided into a double-muscled strain and a dual-purpose strain, each with a separate herd book. The DMBB breed is the most important breed for beef production in Belgium, and it is often used for crossbreeding abroad [3,4], because of its excellent carcass quality [2]. Derno *et al.* [5] reported a variation in energy requirements for maintenance of 10% to 30% because of genetic differences, so that it is not excluded that DMBB and non-DMBB animals have divergent requirements. Hanset *et al.* [6] reported 8% lower maintenance energy requirements for DMBB bulls compared to non-DMBB bulls. However, Vermorel *et al.* [7] found no significant difference in energy expenditure between 10-month old DMBB and non-DMBB bulls. Similar energy requirements for maintenance of double-muscled and non-double-muscled beef steers were obtained when they were scaled to adult and current protein masses [8]. Initially, most nutrient requirements of this young DMBB breed were unknown. In the mean time, energy and protein requirements for growing-finishing beef bulls have been derived [9], but maintenance requirements of DMBB cows are lacking.

The aim of the present experiments was to investigate the energy requirements for maintenance of DMBB beef cows. Maintenance energy requirements can be defined as the daily energy intake that will balance heat production, resulting in no loss or gain of body energy reserves [10].

## 2. Materials and Methods

### 2.1. Animals and Management

Two experiments were conducted, starting in early autumn, and involving 20 and 40 DMBB cows, respectively. Initial age, body weight (BW) and body condition score (BCS), determined as described by Agabriel *et al.* [11], amounted to (mean ± SD) 1319 ± 511 and 1356 ± 512 days, 621 ± 81 and 636 ± 102 kg, and 1.68 ± 0.44 and 2.24 ± 0.50, respectively. Initial age and BW did not differ between experiments (*p* > 0.10), whereas BCS was lower in Exp. 1 than in Exp. 2 (1.7 *vs.* 2.2; *p* < 0.001). Three energy levels (EL) were studied during a restriction period of 112 (Exp. 1) or 140 (Exp. 2) days, respectively: 100% (EL100; Exp. 1 and 2), 70% (EL70; Exp. 1) or 80% (EL80; Exp. 2) of total energy requirements, according the energy evaluation system described by Van Es [12]. Cows were grazing on pasture in similar conditions before the start of both experiments, with grass as the sole component of the diet. They were adapted to confinement and diet during the week prior to the start of the experiments. Within each experiment cows were divided into similar treatment groups based on initial BW, age, BCS and parity, and assigned to one of the energy levels. Protein requirements [13] were always fulfilled. Diets consisted of an appropriate amount of maize silage, individually calculated per animal to realize the programmed EL, supplemented with 0.5 kg per day of a vitamin-mineral premix and urea. Urea was individually fed and top-dressed over the maize silage. The daily amount of urea was calculated so that dietary rumen degradable protein balance (OEB; [13]) was close to 0 g/day. The premix was offered once daily at 1000 h, whereas maize silage and urea were administered in two equal meals at 1000 and 1600 h. Drinking water was always freely available in both experiments.

Cows were confined in uninsulated tie stalls and bedded on sawdust. Monthly outdoor temperature from September to January averaged 14.9, 11.1, 6.8, 3.9 and 3.3 °C, respectively. Cows were weighed in the morning before feeding on two subsequent days at the start and the end of the experiments. BCS was also determined at the start and the end of the experiments. Rectal temperature (RT) was manually measured with a digital thermometer (MT1831, Microlife AG, Widnau, Switzerland) for cows involved in Exp. 2 (EL100 and EL80) at 0800 (T1), 1100 (T2), 1400 (T3) and 1700 h (T4) on 3 days: during the adaptation period on day 2 prior to the start of the experiment (D1), and on days 69 (D2) and 139 (D3). Animals involved in Exp. 1 were part of a larger study [14].

This research was compliant with regulations of the Ethical Committee of the Institute for Agricultural and Fisheries Research (ILVO; approval number 110/2009).

### 2.2. Analytical Procedures

Feeds were sampled every four weeks and chemical composition was determined on a pooled sample of each feed. Moisture was determined by drying at 103 °C [15]. Crude ash was obtained by incineration at 550 °C [16]. Crude protein (N × 6.25) was determined by the Kjeldahl method [17]. Crude fat was extracted with petroleum ether [18]. Neutral detergent fiber (NDF) was analyzed with an Ankom 200 Fiber Analyzer (Ankom Technology, Macedon, NY, USA), using α-amylase and sodium sulphite and expressed on ash-free base [19]. *In vitro* organic matter digestibility was determined using cellulase, to estimate metabolizable energy (ME) and net energy (NE) values as described by De Boever *et al.* [20]. Mean composition, *in vitro* organic matter digestibility and nutritive values of the feeds are shown in Table 1.

**Table 1:** Chemical composition and nutritive value of the feeds.

	Premix		Maize Silage	
	Exp. 1	Exp. 2	Exp. 1	Exp. 2
Dry matter (DM; g/kg)	882	891	343	351
Composition of DM (g/kg)				
Crude protein	100	102	68	72
Crude fat	16	21	33	30
Crude ash	394	383	39	42
NDF	189	157	387	394
*In vitro* organic matter digestibility (%)	84.4	85.1	73.1	69.7
Nutritive value per kg DM				
Metabolizable energy ^1^ (MJ)	4.83	5.27	11.20	10.85
Net energy lactation ^1^ (MJ)	2.51	2.82	6.50	6.23
DVE ^1^ (g)	30	30	47	45
OEB ^1^ (g)	18	20	−35	−30

^1^ Metabolizable and net energy were estimated as described by De Boever *et al.* [20]; DVE: truly absorbed protein in the small intestine; OEB: rumen degradable protein balance as described by Tamminga *et al.* [13].

### 2.3. Statistical Analysis

Animal performances were analyzed using EL and experiment as fixed effects with initial age of the cows as covariate. Rectal temperature (Exp. 2, EL100 and EL80) was analyzed using a design with EL (*n* = 2), day of RT measurement (*n* = 3) and time of the day of RT measurement (*n* = 4) as fixed effects, with day and time of RT measurement as repeated measures.

Metabolizable energy (ME) and NE intakes without correction for initial age of cows were regressed against BW, BW change and initial BCS. Maintenance requirements for ME (MEm) and NE (NEm) were estimated from the regression equation by setting BW gain to zero, using regression analysis. Effect of EL and parity on MEm and NEm requirements were analyzed using an analysis of variance. Absolute MEm and NEm requirements of DMBB cows were compared with energy requirements of beef cows reported in the literature, using an analysis of variance. Furthermore, absolute MEm and NEm requirements from literature data were expressed as a percentage of the mean MEm and NEm requirements of DMBB cows, respectively. Pooled literature data and data from DMBB cows were compared, using an analysis of variance. Statistical analyses were performed using Statsoft Statistica software [21].

Results are presented as least squares means. Treatment effects are presented as significant when *p* ≤ 0.05, and trends are identified at 0.05 < *p* ≤ 0.10.

## 3. Results and Discussion

### 3.1. Effect of Energy Level on Animal Performance

Animal performance was not affected by experiment (*p* > 0.10). Therefore, only the effect of EL on BW and BCS, and energy intake is shown in Table 2. Increasing the level of feed restriction resulted in a decrease of BW and BCS (*p* < 0.001) and an increased daily BW loss (*p* < 0.001). By design, there was a decrease in daily intake of DM, ME and NE (*p* < 0.001), resulting in a lower daily intake of DM, ME and NE per kg BW^0.75^ (*p* < 0.001).

**Table 2:** Effect of energy level on body weight and condition, energy intake and body temperature.

Item ^1^	Energy Level (%)			SEM ^2^	*p*-Value
	100	80	70		
Number of cows	30	20	10		
Body weight (kg)					
Initial weight	631	631	624	6.5	0.954
Final weight	622 ^a^	586 ^b^	556 ^b^	6.2	0.002
Daily gain	−0.07 ^a^	−0.33 ^b^	−0.60 ^c^	0.015	<0.001
Body condition score					
Initial BCS	1.9	1.9	2.1	0.05	0.835
Final BCS	2.0 ^a^	1.6 ^b^	1.2 ^c^	0.04	<0.001
Dry matter intake					
kg/day	6.8 ^a^	5.4 ^b^	4.8 ^b^	0.10	<0.001
g/BW^0.75^/day	54.5 ^a^	44.5 ^b^	40.4 ^b^	0.85	<0.001
ME intake					
MJ/day	70.6 ^a^	52.9 ^b^	49.2 ^b^	0.52	<0.001
MJ/BW^0.75^/day	0.55 ^a^	0.45 ^b^	0.40 ^c^	0.003	<0.001
NE intake					
MJ/day	40.9 ^a^	32.5 ^b^	28.4 ^b^	0.55	<0.001
MJ/BW^0.75^/day	0.33 ^a^	0.27 ^b^	0.24 ^b^	0.005	<0.001
Body temperature (°C)	38.3	38.3	10	0.10	0.938

^1^ BCS: body condition score; ME: metabolizable energy; NE: net energy; ^2^ SEM: standard error of the mean. ^a^, ^b^, ^c^ values within rows with different superscripts differ significantly (*p* < 0.05).

It has been assumed that beef cows can efficiently mobilize and restore body reserve tissues, when feed restriction is followed by an abundant feed supply [22,23]. The effect of an energy restriction on performance of DMBB cows has been reported previously [24].

The metabolism of nutrients generates heat, which contributes to temperature homeostasis. Body temperature of DMBB cows was similar for EL100 and EL80 (*p* > 0.10). However, RT was significantly affected by day (*p* = 0.012) and time of RT measurement (*p* < 0.001), with an interaction between day and time of RT measurement (*p* < 0.001). Temperatures at the start (D1, 38.5 °C) and halfway through the experiment (D2, 38.4 °C) were not different, but RT at D1 was higher compared to RT at D3 (38.1 °C, *p* = 0.010), whereas RT at D2 tended to be higher compared to RT at D3 (*p* = 0.091). Rectal temperatures at T1 (38.1 °C) were lower (*p* < 0.001) than RT measured at T2 (38.4 °C), T3 (38.5 °C) and T4 (38.5 °C), whereas RT at T2, T3 and T4 did not differ (*p* > 0. 10). Mader *et al.* [25] fed Hereford steers *ad lib* or at 90% of *ad lib* intake and found a lower RT (*p* < 0.05) at 0800 and 1600 h and for the entire 4-d test period for steers fed at the lower intake level. Feed deprivation for eight days in sheep and goats also resulted in a lower RT compared to the lowest RT recorded during the baseline period [26]. All in all, RT of DMBB cows was within the normal range for beef cattle [27]. The daily variation in RT in the current experiment is in accordance with the circadian rhythm [28]. The significant decrease of RT towards the end of the experiment may be an effective strategy to save energy, when feed availability is restricted (EL100 as well as EL80).

### 3.2. Energy Requirements for Maintenance

BCS did not exert a significant contribution to energy intake in this study. Therefore, BCS was omitted from the statistical analysis. Maintenance requirements of DMBB cows were neither affected by BCS. This is not in accordance with results of Birnie *et al.* [29], who found that fasting heat production was significantly higher for dairy cows with a low BCS. Furthermore, the higher fasting heat production of thin cows [29] agrees with the higher maintenance requirements of thinner cows [30]. DMBB cows are characterized by their leanness, while fat cows had a BCS, which was more than 3 times the BCS of the thin cows in the experiment of Birnie *et al.* [29].

Regressing daily intake of ME and NE (MJ/kg BW^0.75^/d) on BW and daily BW change (kg/d) resulted in a significant relationship:

ME = 19.227 + 0.0828 BW + 38.911 BW change; *R*^2^ = 0.764, *p* < 0.001; RSD = 5.45 NE = 10.573 + 0.0493 BW + 21.292 BW change; *R*^2^ = 0.579, *p* < 0.001; RSD = 4.72

Setting BW change to zero resulted in MEm and NEm (±SE) of 0.569 ± 0.001 and 0.332 ± 0.001 MJ per kg BW^0.75^, respectively. Maintenance requirements per kg BW^0.75^ of 0.332 MJ for DMBB cows in the current experiment were 35% lower than 0.507 MJ reported for DMBB bulls [9]. Cows fed EL100, EL80 or EL70 showed similar MEm (*p* = 0. 784) and NEm (*p* = 0.369) requirements (Table 3), indicating that maintenance requirements were not affected by plane of nutrition in the present study. However, Birkelo *et al.* [31] found that an increased plane of nutrition in Hereford steers increased fasting heat production and maintenance energy requirements. Fox *et al.* [32] reported that maintenance requirements may also be affected by previous plane of nutrition. Agnew and Yan [33] concluded that it seems unlikely that fasting greatly influences heat production. These authors mentioned that fasting after a long period of restricted nutrition can induce metabolic disorders, such as hypoglycaemia. However, similar blood glucose concentrations at the end of Exp. 1 have been reported previously [14].

Maintenance ME requirements were slightly but significantly higher for primiparous cows compared to older cows (*p* < 0.001), whereas NEm was slightly but significantly lower for second-calf cows compared to other parities (*p* < 0.001). Although the results were statistically significant, due to a small variance, the difference is small (<1%) and may hardly be nutritionally relevant. The effect of parity on maintenance energy requirements of beef cows is scarcely reported in the literature. However, this result is in line with an increasing body fat content in DMBB cows with advancing maturity [34] and the fact that maintenance energy requirements are lower for adipose tissue than for lean tissue [30].

**Table 3:** Effect of energy level and parity on energy requirement for maintenance (MJ/kg BW^0.75^).

	Metabolizable Energy	Net Energy
Energy level (%)		
100	0.569	0.332
80	0.569	0.332
70	0.569	0.331
*p*-value	0.784	0.369
Parity		
1	0.571 ^a^	0.332 ^a^
2	0.568 ^b^	0.331 ^b^
3 and more	0.568 ^b^	0.332 ^a^
*p*-value	<0.001	<0.001
All data	0.569	0.332
SEM ^1^	0.0002	0.0001

^1^ SEM: standard error of the mean; ^a^, ^b^ values within columns with different superscripts differ significantly (*p* < 0.05).

**Table 4:** Comparison of metabolizable (MEm) or net energy (NEm) requirements (MJ/kg BW^0.75^) for maintenance of beef cows of different genotypes.

Genotype	MEm	Reference	Genotype	NEm	Reference
Angus-Hereford crossbreds	0.534	[30]	Angus	0.304	[42]
Angus-Hereford crossbreds	0.544	[35]	Not specified	0.322	[43]
Charolais crossbreds	0.565	[35]	Angus, Exp. 1	0.373	[44]
Simmental crossbreds	0.699	[35]	Angus, Exp. 2	0.389	[44]
Angus	0.418	[36]	Angus, Exp. 3	0.378	[44]
Hereford	0.452	[36]			
Angus	0.656	[37]			
Crossbreds, low milk yield	0.556	[38]			
Crossbreds, moderate milk yield	0.636	[38]			
Crossbreds, low milk yield	0.615	[38]			
Angus	0.433	[39]			
Simmental	0.517	[39]			
Charolais	0.490	[40]			
Angus-Hereford crossbreds	0.503	[41]			
					
Mean (*n* = 14)	0.544		Mean (*n* = 5)	0.353	
SEM	0.023		SEM	0.017	
DMBB (current experiment)	0.569		DMBB	0.332	

The present study revealed that MEm and NEm requirements of DMBB cows were within the range of maintenance requirements reported in the literature for beef cows with other genotypes (Table 4). Maintenance requirements reported by Thompson *et al.* [30], Ferrell and Jenkins [35], Montaño-Bermudez *et al.* [38], Laurenz *et al.* [39], Buskirk *et al.* [42] and NRC [43] differed from DMBB cows by less than 10%, whereas results from other experiments were divergent from our findings by more than 10%. Table 4 shows a range in MEm from 0.418 to 0.699 MJ/kg BW^0.75^, the latter being 68% higher than the lowest value. This variation is nearly the double of the variation of 10% to 30% due to genetic differences, reported by Derno *et al.* [5]. It may be clear that a wide range of animal factors, such as genotype, and environmental factors can influence maintenance energy expenditure [32]. Even the technique to determine maintenance requirements is variable: zero BW change [30,35,36,37,38,44], zero energy retention [39], calorimetry chamber [40] or respiration chamber [41]. Furthermore, extra activity for walking may increase maintenance energy requirements. Locomotion was restricted in our experiments because cows were confined in tie stalls, but maintenance energy requirements on pasture can be increased by 25%–50% [45].

Mean MEm and NEm requirements of DMBB cows amounted to 105% and 94% of the mean MEm or NEm requirements of other genotypes, respectively. Maintenance ME requirements of DMBB cows were higher than those reported in the literature (*p* = 0.021), whereas NEm requirements of DMBB cows were lower (*p* < 0.001). Pooling literature data (Table 4; *n* = 19) with individual results of DMBB cows showed that requirements were 1.5% higher for DMBB cows (*p* > 0.10). Both approaches demonstrate that maintenance energy requirements of DMBB cows are within the range reported in the literature for beef cows.

Double-muscled cattle are characterized by more fast-glycolytic fibers than non-double-muscled animals [2]. Protein turnover of glycolytic fibers is lower than in oxidative fibers [46]. Protein turnover may contribute to about 15% of energy expenditure [47], so that maintenance energy requirements of DMBB cows may be lower than in other genotypes. Furthermore, double-muscled cattle have smaller organs [2], and in general, the mass of organs is highly correlated with energy expenditure [48]. This is another argument to assume lower maintenance energy requirements for double-muscled cattle. Hanset *et al.* [6] reported 8% lower maintenance energy requirements of DMBB bulls in comparison with those of non-DMBB bulls, but it is not clear if the difference was significant. Maintenance energy requirements of DMBB cows are very similar to the mean requirements of other beef genotypes (Table 4). The reducing effect due to a lower protein turnover may be counterbalanced by the higher muscle mass in DMBB animals, and the fact that lean tissue is relatively more metabolically active than fat tissue [30,49]. The similar requirements of DMBB cows and cows of other breeds is in accordance with the findings of Vermorel *et al.* [7], who found no significant difference in energy expenditure between 10-month old DMBB and non-DMBB bulls. Thornton *et al.* [50] found that Red Angus steers, sired by bulls with a lower MEm, had more type I myofibers in the biceps femoris muscles than steers sired by bulls with a higher MEm. Furthermore, steers sired by bulls with a higher MEm resulted in more type IIb fibers compared to steers sired by bulls with a lower MEm. Consequently, the contrasting results of Bergen [46] and Thornton *et al.* [50] showed that the effect of myofiber type on maintenance energy requirements is not equivocal.

## 4. Conclusions

Maintenance energy requirement of DMBB beef cows correspond to the mean requirements of other beef genotypes. Feeding level did not affect maintenance requirements, whereas there was a significant effect of parity. However, the small numerical differences between parities may hardly be nutritionally relevant.
